# Detection of homozygosity and heterozygosity regions in mediterranean sheep breeds revealed by high-density SNP array

**DOI:** 10.1093/jas/skag014

**Published:** 2026-01-20

**Authors:** Federica Carta, Giorgio Chessari, Maria Teresa Sardina, Silvia Riggio, Gabriele Senczuk, Alberto Cesarani, Andrea Criscione, Salvatore Mastrangelo

**Affiliations:** Dipartimento di Scienze Agrarie, Alimentari, e Forestali, Università degli Studi di Palermo, Palermo, 90128, Italy; Dipartimento di Agricoltura, Alimentazione e Ambiente, Università degli Studi di Catania, Catania, 95123, Italy; Dipartimento di Scienze Agrarie, Alimentari, e Forestali, Università degli Studi di Palermo, Palermo, 90128, Italy; Dipartimento di Scienze Agrarie, Alimentari, e Forestali, Università degli Studi di Palermo, Palermo, 90128, Italy; Dipartimento di Agricoltura, Ambiante e Alimenti, Università del Molise, Campobasso, 86100, Italy; Dipartimento di Agraria, Università degli Studi di Sassari, Sassari, 07100, Italy; Department of Animal and Dairy Science, University of Georgia, Athens, 30602, United States; Dipartimento di Agricoltura, Alimentazione e Ambiente, Università degli Studi di Catania, Catania, 95123, Italy; Dipartimento di Scienze Agrarie, Alimentari, e Forestali, Università degli Studi di Palermo, Palermo, 90128, Italy

**Keywords:** runs of homozygosity, heterozygosity-rich regions, sheep, genetic diversity, Illumina Ovine SNP600K SNP array

## Abstract

Genome-wide studies in livestock have become essential tools for investigating genetic diversity, population structure, and adaptive evolution. By leveraging high-density single nucleotide polymorphism (SNP) arrays, researchers can identify genomic regions under selection and trace the demographic history of breeds. Among the most informative genomic features are Runs of Homozygosity (ROH), which reflect inbreeding levels and historical population dynamics, and Heterozygosity-Rich Regions (HRR), which may indicate loci under balancing selection and contribute to important functional traits. In this study, we investigated the ROH and HRR patterns in four different Mediterranean sheep breeds (Barbaresca—BAR, *n* = 48; Noticiana—NOT, *n* = 48; Valle del Belice—VDB, *n* = 142; and Sarda—SAR, *n* = 144) genotyped using the Illumina Ovine SNP600K array. The population structure analysis revealed a distinct separation among the four breeds, likely due to differences in breeding areas or management. Clear differences in ROH and HRR patterns were also observed. The endangered breeds (NOT and BAR) showed a higher mean number of ROH per individual (92.38 and 83.71, respectively) compared to SAR (60.38) and VDB (58.49). A total of 12 ROH islands, ranged from 0.13 to 2.83 Mb, have been detected. These genomic regions mapped genes associated with economically important traits, such as reproduction (*ZDHHC21*), milk (*HERC3* and *HERC6*) and meat (*ABCG2, PKD2, LAP3, NCAPG*, and *SPP1*) production, and body size (*LCORL*). Regarding the HRR, the mean number of segments for individuals ranged from 4.65 (BAR) to 6.50 (VDB), and over 52% of these were shorter than 150 kb. The 16 HRR islands mapped genes related to reproduction (*CAPSPERB* and *TC2N*) and climate adaptation (*VPS13B*). Our results showed the usefulness of ROH and HRR for investigating genomic regions harboring genes associated with important traits that are consistent with the phenotypic characteristics of the investigated breeds, which present differences in both morphology and production traits and show excellent adaptability to the local environments. These findings may help in designing effective breeding or conservation programs for these sheep breeds.

## Teaser

Runs of homozygosity (ROH) and heterozygosity-rich regions (HRR) were analyzed in four Mediterranean sheep breeds using high-density SNP data. Differences in ROH and HRR patterns reflected breed-specific characteristics. Candidate genes associated with production traits and climate adaptation were identified.

## Introduction

High-throughput and cost-effective genotyping techniques in parallel with statistical methods have made it easier to characterize the livestock genomic architecture, offering insights into the evolutionary history of breeds and identifying candidate genes for adaptation, production, and disease resistance (Gurgul et al. 2014).

Runs of homozygosity (ROH) are uninterrupted homozygous ­regions within the genome, often resulting from the matings of genetically related individuals which transmit identical haplotypes to their descendants (Gibson et al. 2006). The heritable nature of these homozygous segments makes them a useful tool capable of providing information about the demographic evolution of a population over time (Peripolli et al. 2017). The ROH occurrence and distribution in the genome are influenced by several factors, such as natural and artificial selection, which determine a non-random pattern of autozygotic segments with many markers within ROH showing high frequencies in populations (i.e. the so-called “ROH islands”) (Gorssen et al. 2021). Examining ROH islands has become a highly efficient method for pinpointing genomic regions subjected to selective pressures and the variants shared between individuals that are directly or indirectly related to the population’s phenotype (Pegolo et al. 2025). More recently, livestock research has focused attention on heterozygosity sequences, which refer to regions of contiguous single heterozygous nucleotides found between homologous chromosomes in diploid organisms and can provide information about populations’ heterozygous state and distribution (Williams et al. 2016). Runs of heterozygosity cannot be defined as true runs but rather as heterozygosity-rich regions (HRR) (Marras et al. 2018; Biscarini et al. 2020), and are not as well described in the literature as ROH are. These regions can vary in length, ranging from just a few consecutive markers to long segments encompassing multiple genes (Chessari et al. 2024). The HRR could be associated with survival rate, fertility, and other fitness traits (Selli et al. 2021). Moreover, the study of these regions can shed light on possible phenomena related to balancing selection, introgression, and hypervariable regions (Mulim et al. 2024).

The ovine species is raised worldwide under a variety of environmental conditions. This species displays a range of morphological characteristics that are hypothesized to have arisen as adaptation to the specific ecological conditions of their respective habitats (Kalds et al. 2022). Over the decades, sheep breeds have developed unique genetic characteristics that make them particularly suited to extreme environmental conditions, such as high temperatures and drought (Joy et al. 2020; McManus et al. 2020). In contrast to other major livestock species, locally adapted sheep breeds have primarily evolved in challenging environments, and they are expected to perform better and withstand the impacts of climate change more effectively than cosmopolitan breeds, which often struggle under similar conditions (Wanjala et al. 2025). Compared to other livestock species, there is also limited use of artificial insemination to transfer sheep genetics across environments, which further reinforces their local adaptation. Considering the significant phenotypic diversity seen in native sheep breeds and their strong influence from natural selection, ecological factors have mainly shaped the divergence and evolutionary adaptation of these genetic resources (Lv et al. 2014).

In this context, Southern Italian sheep breeds represent a valuable case study. Sarda and Valle del Belìce are among the most economically important Italian dairy sheep breeds reared on the islands of Sardinia and Sicily, respectively (Cesarani et al. 2023). Furthermore, the sheep species in Southern Italy is represented by several autochthonous breeds with a small census, exhibiting differences in both morphology and productive traits and possessing excellent adaptability to local environments (Tolone et al. 2012). In particular, the Barbaresca and Noticiana breeds are usually raised in marginal areas of Sicily thanks to their good adaptive traits and rusticity, thus representing an important genetic resource for present and future needs (Mastrangelo et al. 2017a; Chessari et al. 2023).

In this study, we aimed to investigate the distribution and patterns of ROH and HRR in these local breeds to understand the molecular basis of their adaptive and production traits. We employed the 600K HD BeadChip that, thanks to its high marker density, allows for highly accurate and in-depth results, providing a particularly detailed genetic characterization, making this research particularly valuable in filling the existing gap in understanding genetic diversity within these breeds.

## Material and methods

All experimental procedures and sampling were approved by the Bioethics Committee of the University of Palermo: protocol code UNPA-CLE–98597. Blood samples were collected in compliance with the European rules (Council Regulation [EC] No. 1/2005 and Council Regulation [EC] No. 1099/2009) during routine health controls by the public veterinary service. The authors confirm that they have followed EU standards for the protection of animals used for scientific purposes.

### Sampling and DNA extraction

DNA samples were collected from 334 animals (males and females) representing three distinct sheep breeds: Barbaresca (BAR; *n* = 48), Valle del Belice (VDB; *n* = 142), and Sarda (SAR; *n* = 144). The sampling took place across various farms located on the islands of Sicily and Sardinia. The DNA of BAR and VDB was extracted from whole blood samples collected from the animals’ jugular vein using vacutainers containing EDTA at pH 8.0 as an anticoagulant. For DNA extraction, an optimized salting-out protocol was used starting from 1 mL of whole blood. The DNA of SAR was instead extracted from saliva swabs using the MagMax CORE Nucleic Acid Purification Kit from Applied BioSystems™.

### Genotyping and data management

All samples were genotyped using the Illumina Ovine 600K array, which contains 606,006 raw Single nucleotide polymorphism (SNP) markers, providing full coverage of the sheep genome (Illumina, San Diego, California, United States). Moreover, the high-density genotyping data of Noticiana sheep breed (NOT; *n* = 48) were retrieved from a previous study (Chessari et al. 2023). The merged dataset was updated to the ARS-UI_Ramb_v2.0 version of the assembled sheep genome for chromosomal coordinates, positions and SNP names. PLINK v1.9 (Chang et al. 2015) was used to filter the data and perform quality control. After ­excluding unmapped SNPs and markers on sex chromosomes, the following quality parameters were applied: a minimum minor allele frequency of 0.01, a genotype call rate per SNP greater than 0.95, and an individual call rate greater than 0.90. This resulted in a final dataset of 465,236 biallelic variants and 372 sheep.

### Genetic diversity indices and population structure analyses

The four breeds were investigated for genetic diversity indices, including observed (H_O_) and expected (H_E_) heterozygosity, inbreeding coefficient (F_IS_), and average minor allele frequencies (MAF). All indices were calculated using PLINK v1.9 (Chang et al. 2015) software.

To explore individuals’ relationships, the dataset was pruned by setting a window size of 50 kb, a step size of 10 variants, and a pairwise *r^2^* threshold of 0.5 in PLINK v1.9, saving 298,626 SNPs. The multidimensional scaling (MDS) analysis was performed based on pairwise identity-by-state (IBS) distances among individuals using PLINK v1.9, while a neighbor-joining tree was built as 1—IBS and visualized using SplitsTree v4.14.8 (Huson and Bryant 2006). The analysis of genomic structure was performed with the ADMIXTURE v1.3.0 software (Alexander et al. 2009) using the unsupervised model-based clustering algorithm from *K* = 2 to *K* = 6, which estimates the individual ancestry proportions given a *K* number of ancestral populations. The most likely number of clusters was estimated following the cross-validation procedure, whereby the estimated prediction errors are obtained for each *K* value. The estimated matrices were plotted through the R package BITE v1.2.0008 (Milanesi et al. 2017).

### Detection of runs of homozygosity and heterozygosity-rich regions

For ROH and HRR detection, different scenarios were evaluated because of the lack of well-known fixed parameters (Biscarini et al. 2020; Meyermans et al. 2020; Mulim et al. 2024). We conducted ROH and HRR analyses using the sliding window method implemented in the R package detectRUNS v0.9.6 (Biscarini et al. 2018), with the following common parameters: 1) the minimum number of required SNPs was 50 for ROH and 15 for HRR; 2) missing or opposite genotypes were set as zero; 3) the maximum gap between consecutive SNPs was set to 1 Mb; 4) the minimum length was set to 1 Mb for ROH and 100 kb for HRR; 5) a sliding window of 50 SNPs was used for ROH detection, while 15 SNPs for HRR detection; 6) the minimum SNP density was set to one SNP every 100 kb; and 7) the threshold to classify a SNP within ROH was set to 0.05.

ROH and HRR segments were placed into five different classes of length according to their total length using the nomenclature of Kirin et al. (2010) and Ferenčaković et al. (2013): 1–2, 2–4, 4–8, 8–16, and >16 Mb for ROH, <150, 150–200, 200–300, 300–400 and >400 kb for HRR. In both cases, the following parameters were calculated: the mean number of ROH/HRR per individual (N_ROH_/N_HRR_) as well as the average length of ROH/HRR in Mb per individual (L_ROH_/L_HRR_); in addition, the total length of the genome covered by ROH/HRR (S_ROH_/S_HRR_) was evaluated for each individual and divided by the total autosomal genome length covered by SNPs (∼2.4 Gb) in order to evaluate the genomic inbreeding coefficient (F_ROH_) and the degree of diversity (D_HRR_) (Bordonaro et al. 2023), respectively.

### Gene annotation of ROH and HRR islands

To identify the genomic regions that were most associated with ROH/HRR, the percentage of occurrences of SNP in ROH/HRR was estimated by counting the number of times that each SNP appeared in a ROH/HRR and dividing that number by the number of animals per breed. Markers in homozygous (ROH islands) and heterozygous (HRR islands) regions were identified by selecting only the top 0.1% of the SNPs with the highest within-run fixation rate per breed (Selli et al. 2021), determining different thresholds for each breed. Adjacent SNPs with a proportion of ROH/HRR occurrences over the adopted threshold formed the islands. Moreover, an additional threshold of frequency >20% and 30% was implemented to call a ROH and HRR island, respectively (Mastrangelo et al. 2017b; Biscarini et al. 2020). These regions were examined for gene annotation using the Ensembl database according to ARS-UI_Ramb_v2.0 genome assembly (GCA_016772045.1, released 113, accessed on April 15, 2025). Prediction of the variants effect was performed using Variant Effect Predictor (VEP) tool (Clark et al. 2019) for each SNP list generated per breed, by setting a downstream/upstream distance of 1,000 bp. Subsequently, a comprehensive literature review was conducted to investigate the biological function and phenotypes known to be affected by each annotated gene.

## Results

### Genetic diversity indices and population structure analyses

The results about genetic diversity indices are reported in Table S1 (see online supplementary material). Comparable results in terms of H_O_, ranging from 0.306 (NOT) to 0.326 (VDB), were found. The H_E_, on the other hand, showed a different trend among the breeds with values below (BAR and NOT) or above (SAR and VDB) 0.300. Similarly, BAR and NOT were the two breeds with H_O_ values lower than H_E_. The inbreeding coefficient F_IS_ revealed the highest value for NOT breed (0.096 ± 0.054), as confirmed by the highest F_ROH_ (0.100 ± 0.050) (Table 1). Lastly, MAF values ranged from 0.221 (NOT) to 0.244 (VDB), showing similar values among breeds (Table S1—see online supplementary material).

**Table 1:** Descriptive statistics for ROH and HRR investigated using the sliding window detection method. The total number of runs per breed was reported. Values per breed are tabulated by the mean number of ROH/HRR per individual (N_ROH_/N_HRR_), the average length of ROH/HRR in Mb per individual (L_ROH_/L_HRR_), the total length of the genome covered by ROH/HRR (S_ROH_/S_HRR_), the genomic inbreeding coefficient (F_ROH_) and the degree of diversity (D_HRR_). Standard deviation (s.d.) was calculated as well.

		BAR (*n* = 48)	NOT (*n* = 48)	SAR (*n* = 140)	VDB (*n* = 136)
**ROH statistics**	*Total runs*	4,018	4,434	8,453	7,545
	***N*_ROH_ ± s.d.**	83.71 ± 33.79	92.38 ± 34.73	60.38 ± 27.97	58.49 ± 39.34
	***L*_ROH_ ± s.d.**	2.72 ± 0.36	2.58 ± 0.37	2.35 ± 0.36	2.34 ± 0.45
	***S*_ROH_ ± s.d.**	232.01 ± 110.81	247.58 ± 120.33	146.52 ± 83.99	146.01 ± 109.92
	***F*_ROH_ ± s.d.**	0.09 ± 0.04	0.10 ± 0.05	0.06 ± 0.03	0.06 ± 0.04
**HRR statistics**	*Total runs*	214	274	790	884
	***N*_HRR_ ± s.d.**	4.65 ± 1.79	5.71 ± 2.45	5.64 ± 2.15	6.50 ± 3.19
	***L*_HRR_ ± s.d.**	0.17 ± 0.04	0.06 ± 0.02	0.15 ± 0.02	0.15 ± 0.03
	***S*_HRR_ ± s.d.**	0.79 ± 0.35	0.92 ± 0.39	0.86 ± 0.34	1.00 ± 0.50
	***D*_HRR_ ± s.d.**	0.00032 ± 0.00014	0.00037 ± 0.00016	0.00035 ± 0.00014	0.00041 ± 0.00020

BAR = Barbaresca; NOT = Noticiana; SAR = Sarda; VDB = Valle del Belice; ROH = Runs of Homozygosity; HRR = Heterozygosity-Rich Regions.

**Figure 1:**
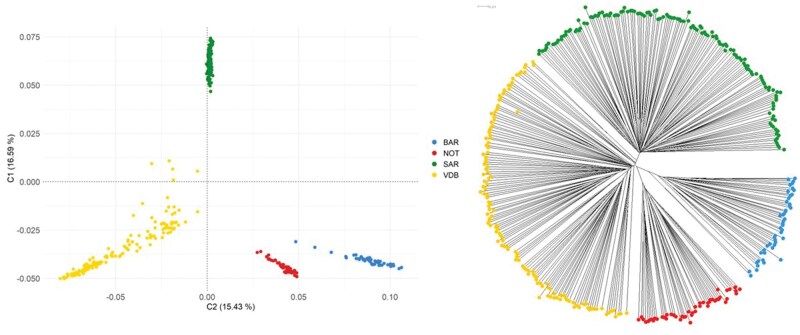
Genetic relationship based on (a) the multidimensional scaling (MDS) analysis and (b) Neighbor-Joining tree based on the IBS parameter. BAR = Barbaresca; NOT = Noticiana; SAR = Sarda; VDB = Valle del Belice.

Both the MDS and the individual-based neighbor-joining tree derived from IBS distances did not reveal clear substructures within breeds (Figure 1). Instead, they highlighted a high degree of compactness and genetic uniformity. Moreover, the VDB individuals formed more dispersed cluster, which is typical of breeds that have experienced admixture with other breeds. Slightly different results were observed in the admixture analysis. Although each breed generally exhibited a distinct genetic identity and structure at *K* = 4, the lowest cross-validation error indicated that the most suitable number of hypothetical ancestral populations was *K* = 6. At this level, clear substructures emerged within the VDB breed, already apparent at *K* = 5, and within NOT breed. The linear plots illustrating admixture proportions from *K* = 2 to *K* = 6 are presented in Figure S1 (see online supplementary material for a color ­version of this figure).

### Run of homozygosity and heterozygosity-rich regions patterns

**Figure 2:**
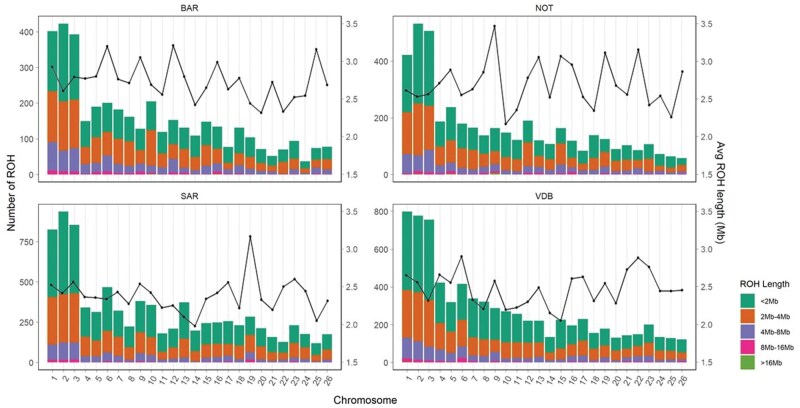
Runs of homozygosity (ROH) counts and distribution per chromosome and breed (left-y axis). The average length of runs of homozygosity in Mb is also reported into the right-y axis. The bar plots show five different length classes. BAR = Barbaresca; NOT = Noticiana; SAR = Sarda; VDB = Valle del Belice.

ROH analysis revealed a total of 24,450 runs, distributed across the 26 autosomes (Figure 2). The highest number of ROH was detected on OAR02 for BAR (423), NOT (533), and SAR (937), while for VDB, the highest number was observed on OAR01 (799). The ROH count per individual ranged from 2 to 236, whereas the N_ROH_ ranged from 92.38 (± 34.73) in NOT to 58.49 (± 39.34) in VDB (Table 1). The L_ROH_ values showed similar mean lengths across all breeds, averaging around 2.50 Mb. Almost all segments were classified as short runs under 4 Mb (85.39% of the total), while only 15 ROH were classified as long segments (> 16 Mb). On average, SAR and VDB had the lowest genome coverage in ROH (S_ROH_), with consistent F_ROH_ values under 0.12 for most of the individuals (Figure S2—see online supplementary material for a color version of this figure).

**Figure 3:**
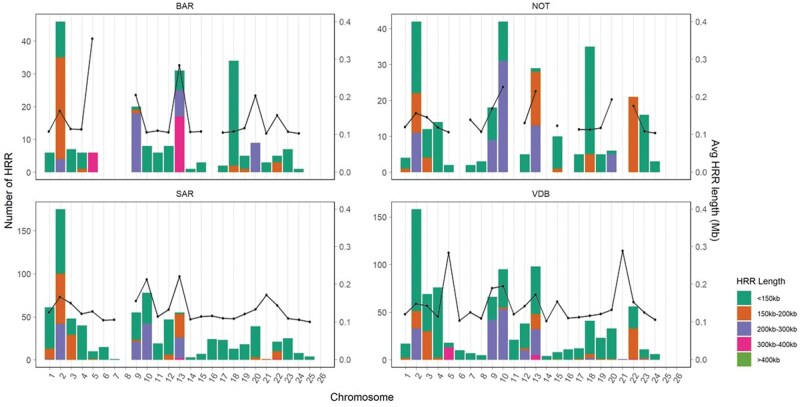
Heterozygosity-rich regions (HRR) counts and distribution per chromosome and breed (left-y axis). The average length of heterozygosity-rich regions in Mb is also reported into the right-y axis. The bar plots show five different length classes. BAR = Barbaresca; NOT = Noticiana; SAR = Sarda; VDB = Valle del Belice.

HRR investigation revealed a total of 2,162 segments, with an average number of regions per sample equal to 4.65 ± 1.79 for BAR, 5.71 ± 2.45 for NOT, 5.64 ± 2.15 for SAR, and 6.50 ± 3.19 for VDB (Table 1). These segments were not evenly distributed across all chromosomes (Figure 3): the highest number of HRR was found in OAR02 for all breeds. Additionally, no HRR segments greater than 1.5 Mb were identified. Notably, at least 52.80% of the segments were shorter than 150 kb. The diversity index estimated from HRR segments (D_HRR_) reported similar results, highlighting comparable heterogeneity between breeds (Figure S3—see online supplementary material for a color version of this figure).

### ROH and HRR islands investigation

**Figure 4:**
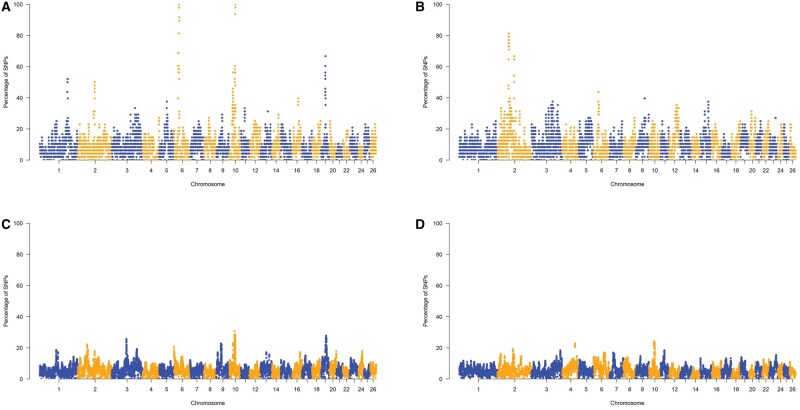
Manhattan plots of frequency of a single nucleotide polymorphism (SNP) in run of homozygosity islands in (A) Barbaresca, (B) Noticiana, (C) Sarda and (D) Valle del Belice sheep breeds.

**Figure 5:**
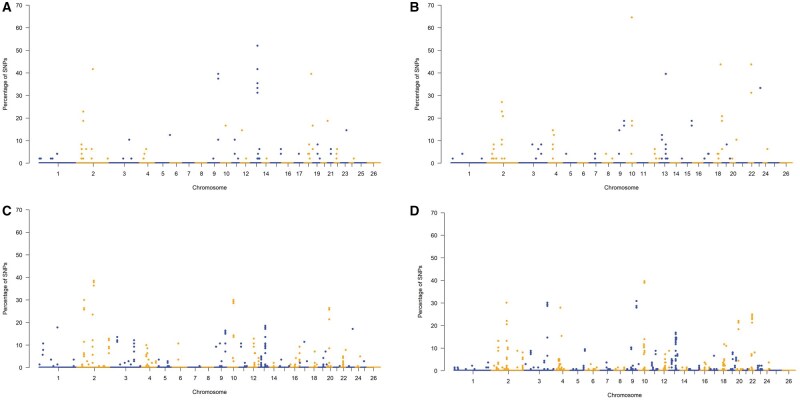
Manhattan plots of frequency of a single nucleotide polymorphism (SNP) in heterozygosity-rich regions in (A) Barbaresca, (B) Noticiana, (C) Sarda and (D) Valle del Belice sheep breeds.

Additional analyses were performed on ROH and HRR patterns to investigate repeated autozygous and heterozygous segments within breeds (ROH and HRR islands). In total, this study identified 12 ROH islands and 16 HRR islands (Tables 2 and 3). Figures 4 and 5 showed the frequency of SNPs within ROH or HRR across the autosomal chromosomes, revealing highly homozygous and heterozygous genomic regions. ROH islands ranged from a maximum SNP fixation index of 100% to a minimum of 97.92% in BAR, from 81.25% to 72.92% in NOT, from 30.71% to 25.71% in SAR, and from 24.26% to 21.32% in VDB, including a total of 466, 525, 644, and 532 biallelic markers, respectively. The largest ROH island was found in NOT, on OAR02, with a length of 2.86 Mb. The region located on OAR10 (42.32–43.39 Mb) overlapped in three of the four sheep breeds (BAR, SAR, and VDB), whereas a region on OAR10 (37.39–38.79 Mb) was shared between SAR and VDB. The SAR breed showed the highest number of ROH islands.

**Table 2:** Run of homozygosity (ROH) islands identified within each breed. The chromosome (OAR), the positions of the genomic regions (in base pairs, bp), the number of single nucleotide polymorphisms (SNPs), the average percentage of fixation (Fix%) and the annotated genes within each ROH island are reported. Common ROH islands for two or more breeds are colored in different shades of grey. In bold those genes affected by variant with a moderate impact according to Variant Effect Predictor analysis.

Breed	OAR	Start bp	End bp	SNPs	Fix%	Genes
**BAR**	6	36 713 446	39 067 495	291	99.96%	*HERC3, NAP1L5, PYURF, ENSOARG00020023567*, ***HERC6*** *, PPM1K, ABCG2*, ***PKD2***, ***SPP1, MEPE*** *, IBSP*, ***LAP3***, ***FAM184B*** *, DCAF16*, ***NCAPG***, ***LCORL*** *, ENSOARG00020029550, ENSOARG00020032248*
	**10**	42 322 026	43 925 475	175	99.96%	*ENSOARG00020035296, ENSOARG00020035750*
**NOT**	2	82 601 893	85 428 011	525	75.74%	*ENSOARG00020028478, ENSOARG00020033455, ENSOARG00020032799, NFIB, ENSOARG00020040248, ZDHHC21, ENSOARG00020030911, ENSOARG00020034846, CER1*, ***FREM1*** *, ENSOARG00020034967, ENSOARG00020037234, TTC39B, ENSOARG00020040142, SNAPC3, PSIP1, ENSOARG00020037607*, ***CCDC17*** *1, ENSOARG00020032888, BNC2*
**SAR**	3	106 277 679	106 854 930	23	25.71%	*FBLN7, ZC3H8, ENSOARG00020035986, ZC3H6, ENSOARG00020036183, ENSOARG00020026712*
	**10**	37 074 391	38 323 145	130	27.74%	*ENSOARG00020030504, ENSOARG00020035296*
	**10**	42 322 026	43 393 956	108	27.19%	*ENSOARG00020035296, ENSOARG00020035750*
	**19**	35 944 655	36 074 417	21	25.82%	*MAGI1, ENSOARG00020033686*
	**19**	36 582 133	37 265 170	148	26.77%	*ENSOARG00020012610, ENSOARG00020030976, ENSOARG00020038383, ENSOARG00020030184, ENSOARG00020026999, ADAMTS9, ENSOARG00020030753, PRICKLE2*
	**19**	37 986 438	39 058 275	214	25.77%	*SYNPR, ENSOARG00020032605, CADPS, FEZF2*
**VDB**	4	93 280 238	94 696 523	307	21.93%	*ENSOARG00020039523, ZNF800*, ***GCC1*** *, ARF5, FSCN3*, ***PAX4*** *, SND1, LRRC4, ENSOARG00020035180, ENSOARG00020037327, ENSOARG00020030512, ENSOARG00020036546, ENSOARG00020029720, MIR129-1, ENSOARG00020040526*, ***LEP*** *, ENSOARG00020032751*, ***RBM28*** *, PRRT4, ENSOARG00020027789*, ***IMPDH1, HILPDA*** *, GARIN1A, CALU, OPN1SW*, ***CCDC136*** *, FLNC, ATP6V1F, ATP6V1FNB*, ***KCP***, ***IRF5*** *, TNPO3*
	**10**	37 396 295	38 863 812	114	22.42%	*ENSOARG00020030504*
	**10**	42 322 720	43 393 956	111	22.69%	*ENSOARG00020035296, ENSOARG00020035750*

BAR = Barbaresca; NOT = Noticiana; SAR = Sarda; VDB = Valle del Belice.

**Table 3:** Heterozygosity rich region (HRR) islands identified within each breed. The chromosome (OAR), the positions of the genomic regions (in base pairs, bp), the number of single nucleotide polymorphisms (SNPs), the average percentage of fixation (Fix%) and the annotated genes within each HRR island are reported. Common HRR islands for two or more breeds are colored in different shades of grey. In bold those genes affected by variant with a moderate impact according to Variant Effect Predictor analysis.

Breed	OAR	Start bp	End bp	SNPs	Fix%	Genes
**BAR**	2	123 468 577	123 637 801	24	41.67%	*FSIP2*
	**9**	77 728 073	77 935 397	18	38.89%	*VPS13B, ENSOARG00020009652*
	**13**	49 229 596	49 594 105	43	47.67%	*–*
	**18**	54 247 130	54 348 566	19	39.58%	*CATSPERB, TC2N*
**NOT**	10	42 419 945	42 678 764	25	64.58%	*ENSOARG00020035296*
	**13**	49 785 151	49 978 019	19	39.58%	*–*
	**18**	54 247 130	54 348 566	19	43.75%	*CATSPERB, TC2N*
	**22**	22 332 864	22 519 184	24	41.15%	*KCNIP2, ARMH3*
	**23**	41 944 403	42 054 238	20	33.33%	*NDUFV2, ANKRD12, ENSOARG00020030516*
**SAR**	2	51 458 838	51 550 541	12	30.00%	*ZCCHC7*
	**2**	123 468 577	123 637 801	24	38.13%	*FSIP2*
	**10**	42 441 586	42 678 764	23	30.00%	*ENSOARG00020035296*
**VDB**	2	113 906 067	114 026 175	15	30.15%	*NIPA2, CYFIP1, ENSOARG00020028055*
	**3**	171 435 795	171 532 877	14	30.15%	*WASHC3, NUP37, PARPBP*
	**9**	77 754 258	77 935 397	15	30.88%	*VPS13B, ENSOARG00020009652*
	**10**	42 441 586	42 678 764	23	39.58%	*ENSOARG00020035296*

BAR = Barbaresca; NOT = Noticiana; SAR = Sarda; VDB = Valle del Belice.

Maximum SNP fixation percentages for HRR islands were 64.58% in NOT, 52.08% in BAR, 39.71% in VDB and 38.57% in SAR, defined by 107, 104, 67, and 59 markers, respectively. The longest HRR was found in the BAR (OAR13), with a length of 364.51 kb, while the shortest HRR was found in the SAR (OAR02: 51 458 838–51 550 541 bp), with a length of 91.70 kb. We identified regions in four chromosomes (OAR2, OAR09, OAR10, and OAR18) where HRR were frequent in more than one population. The HRR on OAR10, mapping the gene *ENSOARG00020035296*, overlaps among NOT, SAR, and VDB. This region was also reported as ROH island shared among SAR and VDB.

The results of the VEP analysis were visualized as pie charts for each breed and run (Figure S4—see online supplementary material for a color version of this figure), according to their predicted genomic consequences. Furthermore, the genes annotated within each genomic island are presented in Tables 2 and 3. Variant ­annotation using VEP revealed a range of predicted consequences across the detected SNPs, including intergenic, intronic, synonymous, and missense variants. Among these, a notable proportion of missense variants were classified as having a moderate impact on gene function. This category includes single nucleotide changes that alter the amino acid sequence of proteins, potentially affecting protein structure or function, without necessarily causing a complete loss of activity. Genes affected by markers predicted to have a moderate impact on their function are highlighted in bold. In general, a total of 99 genes (93 unique) were identified within ROH islands, whereas only 25 genes (18 unique) were associated with HRR islands.

## Discussion

In the last decades, genomic tools have been used to investigate genomic architecture in local or cosmopolitan sheep breeds (Kijas et al. 2012; Ciani et al. 2014). Exploring ROH and HRR profiles within and across populations sheds light on the selective mechanisms that influence the demographic history of populations and also provides insights into the underlying stochastic processes shaping genome-wide diversity (Ceballos et al. 2018). Currently, an increasing number of studies have shown that the existence of such regions not only provides insights into genome structure but can also be utilized to explore genetic diversity and adaptive evolution. Moreover, the high-density (HD) genotyping arrays are extremely valuable tools for genomic studies due to their comprehensive genome-wide coverage and high analytical throughput (Kranis et al. 2013).

### Genetic indices and relationships

To investigate genetic diversity and population structure, we employed a range of analytical approaches. In genome-wide and biodiversity studies, the assessment of heterozygosity and inbreeding coefficients represents a fundamental step in breed characterization, enabling the identification of structural changes within the breed over time (Leroy 2014). By comparing these indices across different time points, it becomes possible to detect events that may have led to either positive or negative shifts in genetic variation. In our case study, the observed genetic diversity indices were in line with those reported in previous studies. Studies conducted in the early 2010s reported balanced levels of heterozygosity and inbreeding in several Italian breeds, including some Sicilian breeds (e.g. Comisana) that are genetically close to the breeds under investigation in this study (Kijas et al. 2012; Ciani et al. 2015). These findings are still supported by our current analyses, suggesting that ongoing management strategies are effective at not increasing inbreeding. This genetic stability is likely the result of well-implemented breeding programs and sustained collaboration between researchers and breeders. For example, the VDB breed has been the focus of several conservation and improvement programs aimed at reducing inbreeding levels and enhancing productive traits (Mastrangelo et al. 2017b). In a previous study by Ciani et al. (2020), the VDB and SAR breeds exhibited slightly higher values of heterozygosity, as estimated from medium-density SNP panels. Previous research has already demonstrated that the use of higher-density SNP arrays can lead to a reduction in observed diversity indices. Specifically, a higher coverage of markers, such as that provided by high-density arrays, and the design of arrays incorporating a broader range of breeds can yield more accurate estimates (Schaefer et al. 2017). However, increasing panel density may also reduce the proportion of markers that are informative for heterozygosity, potentially leading to a slight decrease in heterozygosity values (Falchi et al. 2024).

The population structure analysis showed a distinct separation among the four breeds, as already highlighted in previous studies (Tolone et al. 2012; Mastrangelo et al. 2017a; Chessari et al. 2023). SAR was the most distinct breed, due to its different origin, while BAR and NOT were positioned close to each other. Additional insights were provided by the admixture analysis, which clearly distinguished the BAR and SAR breeds (Chessari et al. 2023). Moreover, the clustering pattern of the VDB breed is well established and has been confirmed by previous studies (e.g. Ciani et al. 2020). As the number of ancestral clusters increased, NOT displayed internal heterogeneity in the genomic structure of its individuals, creating its own genomic identity. These patterns could be explained by the phylogenetic proximity to other local breeds not included in the present study (e.g. Comisana), as well as by gene flow resulting from the movement of individuals among the numerous farms in the region (Tolone et al. 2012; Chessari et al. 2023).

### Runs of homozygosity investigation

It has been shown that ROH patterns are non-randomly distributed across genomes but reflect the occurrence of demographic events and selection (Peripolli et al. 2017). In particular, the presence of ROH islands, as genomic regions shared among multiple individuals within a breed or species, can be indicative of selective sweeps, whether of natural or artificial origin (Pemberton et al. 2012; Grilz-Seger et al. 2018). In our analysis, clear differences in ROH counts and distribution were observed among the four sheep breeds. The BAR and NOT breeds displayed the highest mean number of ROH and inbreeding coefficients, suggesting comparable levels of inbreeding and pointing to the role of geographic isolation in shaping their genomic profiles (Mastrangelo et al. 2017a; Chessari et al. 2023). Conversely, the two dairy breeds (SAR and VDB) showed similar ROH patterns, which may be attributed to similar selection histories for milk production and the presence of common ancestral genetic components (Tolone et al. 2012). The majority of ROH segments identified in our study were short (< 4 Mb), suggesting that the observed homozygosity is primarily the result of ancient inbreeding events (Howrigan et al. 2011). This pattern is consistent with expectations for populations that have experienced historical bottlenecks or long-term genetic drift without recent intensive inbreeding. Moreover, our findings corroborate those of Falchi et al. (2024), who reported that shorter ROH segments are more readily detected with high-density SNP panels, highlighting the enhanced resolution and sensitivity afforded by such genotyping platforms. Indeed, even if the N_ROH_ is comparable to other studies (Mastrangelo et al. 2014; Mastrangelo et al. 2018), L_ROH_ revealed some differences, leading to the detection of shorter runs.

The use of the high-density SNP array allowed us to thoroughly investigate the ROH islands and led to the identification of genomic regions harboring candidate genes connected to production-related factors. Some regions overlapped among the breeds, as the ROH island on OAR10 (42.32–43.39 Mb) was shared by BAR, SAR, and VDB. This result supports the hypothesis that selection behind some genomic variants may be independent from the productive direction and from the anthropic action on different breeds (Szmatoła et al. 2016).

In the BAR breed, we observed two regions where an average fixation percentage of 99.96% was observed, indicating that some specific alleles have been fixed in the population, presumably due to selection or genetic drift, thus suggesting adaptation to local conditions. The region on OAR06 (36.71–39.07 Mb) has also been identified in several studies on sheep (Al-Mamun et al. 2015; Manunza et al. 2016) and other species (Takasuga 2016). These results suggested that some of the ROH islands are common among different breeds and harbor variants that are undergoing selection. Moreover, a previous study on the same breed, on different samples, and using a medium density-array, identified the identical ROH island (Mastrangelo et al. 2019a). This result supports the robustness of the previous findings and suggests that these regions may consistently harbor genomic variants of interest. Indeed, several genes located in this region have been previously reported to be strongly associated with positive selection in other domestic livestock. For example, *HERC3* gene is associated with immune responses and resistance to disease (Al Kalaldeh et al. 2019), whereas *ABCG2* and *LAP3* with milk yield and composition (Cohen-Zinder et al. 2005). Moreover, *NAP1L5*, *SPP1*, *NCAPG*, and *LCORL* genes are associated with body weight and carcass growth (Lindholm-Perry et al. 2013; Al-Mamun et al. 2015; Ramljak et al. 2024), consistent with the phenotypic characteristics of the BAR breed, a fat-tailed sheep. The region on OAR2 in NOT (at position 82.60–85.43 Mb) overlapped with the homozygosity island already identified in Italian dairy sheep breeds (Mastrangelo et al. 2018). This island has also been described in a previous study by Chessari et al. (2023), with several interesting genes, such as *BNC2* involved in coat pigmentation (Fariello et al. 2014). This island also enclosed *ZDHHC21* and *FREM1* genes, identified as associated with fertility (Kiser et al. 2019; Deng et al. 2022), underscoring the potential functional relevance of this homozygosity island. The ROH islands identified in the SAR breed harbored genes associated with reproduction and growth traits (*ADAMTS9*) (Tang et al. 2019), mammary gland development and milk production traits (*MAGI1*) (Taye et al. 2017), and feed efficiency (*SYNPR*) (Ma et al. 2025). Moreover, the same genomic region here identified on OAR19 was also reported as ROH island in a previous study on SAR breed (Mastrangelo et al. 2019b), using a medium array. In the VDB breed, on OAR04 we found candidate genes involved in milk traits, such as *SND1* and *LRRC4* associated with somatic cell count and milk yield (Singh et al. 2020), and *LEP* gene in milk production (Safina et al. 2021). The *ARF5* gene is a candidate with function in immune responses and overseeing apoptosis and has been identified as undergoing selection in local sheep breeds (Wanjala et al. 2025). *HILPDA* is a small, lipid-droplet-associated protein expressed in several tissues that elevates lipid storage in hepatocytes, adipocytes, and macrophages through directly binding and inhibiting adipose triglyceride lipase and related with body weight in sheep (Khazaei-Koohpar et al. 2024).

Among the variants detected within ROH islands, the moderate-impact missense SNPs were located in coding regions of several genes of clear biological interest, including those previously discussed for their roles in growth, body size, and milk production traits. Although their predicted impact was classified as moderate, the resulting amino acid substitutions could modulate protein activity in ways consistent with breed-specific selection histories. The presence of these missense variants within functionally relevant genes therefore suggests that coding variation may have contributed to historical or ongoing selection, reinforcing the importance of these genomic regions in shaping breed-specific phenotypic differences and production traits.

### Heterozygosity-rich regions investigation

Contrary to ROH islands, heterozygosity-rich regions (HRR) are poorly characterized in livestock, and, to our knowledge, only three studies have been conducted in sheep (Selli et al. 2021; Tsartsianidou et al. 2021; Szmatola et al. 2025), and none of these have used HD data. Using this information into breeding programs can help preserve genetic diversity and enhance resilience (Chessari et al. 2024).

The information in literature regarding the setting of the detection parameters provides a non-standardized framework that affects the results and their comparison (Chessari et al. 2024). Mulim et al. (2024) showed that the minimum number of markers, maximum homozygous markers, and the minimum length size impacted HRR detection. Biscarini et al. (2020) found a significant increase in the number and average size of the detected HRR when increasing numbers of missing and/or homozygous SNPs are allowed. In our study, missing and homozygous SNPs were set to zero. Consequently, HRR shorter than 150 kb constituted the majority regions, and HRR segments greater than 1.5 Mb were not identified. In a recent study on Greek insular goats, most of the HRR were classified in the size class 0.4–0.8 Mb (Tsartsianidou et al. 2024), as well as in local pigs, the majority of the detected HRR (86.55%) exhibited short lengths, ranging from 0.25 to 0.5 Mb (Liu et al. 2024). Both the number and total length of HRR decreased with increasing segment size, consistent with the notion that shorter HRR are more common (Chessari et al. 2024; Szmatola et al. 2025). Very similar results were reported in dog, for which Halvonik et al. (2025) showed that the majority of detected HRR did not exceed 0.5 Mb, with a maximum of 1.4 Mb. We found a low average number of HRR per breed (<7), like other studies in cattle breeds (Williams et al. 2016; Biscarini et al. 2020). A study on Chinese indigenous pigs that used whole-genome re-sequencing data, reported a lower average number per breed (from 1.19 to 16.01) (Chen et al. 2022). In contrast, the highest values have been reported in horse (52.2) (Santos et al. 2021), in pig (∼40) (Ruan et al. 2022), and sheep (28.28 and 139.59) (Selli et al. 2021; Tsartsianidou et al. 2021). These differences may be attributed to the species of interest, to the analyzed breeds, to the different SNP arrays and computational parameters used for HRR detection (Ruan et al. 2022; Chessari et al. 2024). For example, it has been observed that the number of allowed homozygous and missing SNPs influences the total number of runs detected, still not altering the trend among breeds (Fabbri et al. 2025).

The descriptive statistics for HRR reflect the breeding histories and selection that have shaped the genetic architecture of these four sheep breeds, providing additional insights into the heterozygosity patterns. For example, for D_HRR_ coefficients, which quantify the proportion of the genome covered by HRR, the VDB showed the highest values, reflecting the low levels of inbreeding, gene flow, and the presence of admixture, factors that can be advantageous in maintaining adaptability and reducing the risk of inbreeding depression. The BAR breed showed the lowest HRR counts and D_HRR_, showing notably lower levels of heterozygosity within the genome.

None of our HRR regions have been previously identified in sheep. Selli et al. (2021) reported common genomic regions and candidate genes in HRR islands for two or more worldwide sheep populations on chromosomes 1, 8, 13, 21, and 26, whereas Tsartsianidou et al. (2021) on chromosomes 3, 10, 13, and 19, both using the Oar_v4.0 sheep genome assembly. The two studies see chromosomal overlapping for OAR13, but for different regions. The heterozygous-rich segment on chromosome 10 (38.57–39.06 Mb) reported by Tsartsianidou et al. (2021) is mapped near to the HRR shared by BAR, NOT, and SAR breeds in the present study. The presence of favored heterozygosity shared across global breeds and clustered in specific chromosomes could indicate genomic regions under balancing selection (Chessari et al. 2024; Tsartsianidou et al. 2024). In contrast to the previous studies, which used medium-density SNP arrays, our data was obtained using a high-density chip, leading to a more accurate assessment of HRR (Arias et al. 2024). Therefore, it is not possible to compare our findings with other studies since this is the first study examining the pattern of HRR in this species using a high-density array. A previous study demonstrated that analyses of HRR in cattle using 50K and 700K SNP arrays showed overestimated HRR lengths and longer HRR when using the 50K array (Szmatoła et al. 2024). Regarding these findings, several studies evidenced how the density of genomic information influences the results of ROH detection (Purfield et al. 2012; Ferenčaković et al. 2013). This phenomenon most probably occurs during the process of HRR detection and highly influences its accuracy.

We detected HRR islands with candidate genes related to immune response, reproduction, and local adaptation, and therefore associated with natural selection (Chessari et al. 2024). The HRR on OAR02 (shared by BAR and SAR) and ORA18 (shared by BAR and NOT) showed candidate genes associated with an important role in sheep fertility. *FSIP2* is a protein-coding gene that plays an important role in spermatogenesis (Fang et al. 2021). In the context of domestication, the *FSIP2* gene is very interesting because it was previously identified as a gene important for domestication (Cao et al. 2021; Chen et al. 2021) i.e. a gene associated with adaptation rather than production traits (Lukic et al. 2023). However, in NOT sheep, this region has been identified in an ROH island (Chessari et al., 2023). Several studies reported that genomic regions highly heterozygous in one breed can be strictly homozygous in another breed (Selli et al., 2021; Chessari et al., 2024). This aspect underlines the general diversity among breeds, linked to the different breeding programs (Bordonaro et al. 2023). Similar results have been reported by Tsartsianidou et al. (2021) in Mediterranean sheep, showing genes related to sheep domestication in HRR, implying positive selection on heterozygous genotypes. Similarly, the *CATSPERB* gene was associated with spermatogenesis (Wang et al., 2009; Sun et al., 2022), and the *TC2N* in the regulation of follicular maturation in goats (Zhao et al., 2020). Moreover, other HRR showed candidate genes associated with fertility traits, like *ARMH3* (Jayawardana et al., 2023) identified in NOT and *ZCCHC7* (Metodiev et al., 2018) in SAR breeds. We also found a well-known candidate gene, the *VPS13B*, which plays a role for adaptation to hot environments (Amiri Ghanatsaman et al. 2023). In fact, *VPS13B* has been reported under selection in the Mediterranean sheep (Yang et al. 2016) and goats (Serranito et al. 2021). Moreover, the VPS13B protein has a role in the formation and development of adipocytes (Seifert et al. 2011). In fat-tailed sheep, such as BAR sheep, adipocytes, or fat cells, accumulate significantly in the tail region, forming the characteristic fat tail (Zhang et al. 2024). In the HRR on OAR22 of NOT sheep, we identified *NDUFV2*, a gene related to local adaptation (Ben-Jemaa et al. 2021). Finally, on OAR02 for the VDB breed, there are *NIPA2* and *CYFIP1*, candidate genes involved in immune system mechanisms (Vera et al. 2024). The idea that genes related to the immune system could be the best candidate in terms of heterozygosity hotspots is here reinforced. Other studies reported genes with similar function in HRR (Li et al. 2022; Chessari et al. 2024) that could be the result of long-term balancing selection following domestication and may be essential for domesticated sheep. The shared HRR island identified might be mainly related to the breeds’ geography (Chessari et al. 2024) and reflect similar adaptive needs (Chen et al. 2022).

### Shared ROH and HRR within breed

ROH and HRR can occur in the same genomic regions across different breeds (Bordonaro et al. 2023; Chessari et al. 2024; Pegolo et al. 2025), suggesting a nuanced genetic structure where homozygosity and heterozygosity play an important role in the adaptation (Tsartsianidou et al. 2021). An interesting result of our study was the occurrence of ROH and HRR islands within the same genomic region but in different individuals of the same breed. This was observed in SAR and VDB sheep on OAR10 (42 441 586-42 678 764). In both breeds, the coexistence of ROH and HRR islands indicates that this region may be under balancing selection, affecting 30.00% of SAR and 39.58% of VDB individuals, respectively. Conversely, for 27.19% of SAR and 22.69% of VDB individuals, directional selection appears to override balancing forces, resulting in ROH formation in the same region. In this genomic region is located the protein-coding gene *ENSOARG00020035296* that is associated with more than 7,500 variant alleles. The region also encompassed several constrained elements conserved across for 91 eutherian mammalian species. A complex evolutionary scenario could be hypothesized, in which purifying selection limited nucleotide changes and maintained functions of a highly conserved region among species (Cooper et al. 2005; Cooper et al. 2010; Mathur et al. 2023), while a heterozygous state was introduced via gene flow, balancing forces, or deleterious mutations (Kyriazis et al. 2021). The current overlapping between ROH and HRR hotspots may represent snapshots of different timescales in different lineages. Other hypotheses might involve structural variants, such as inversions or duplications, causing apparent heterozygosity artifacts (false HRR islands) in a region subject to variability limitations (Collins et al. 2020).

## Conclusion

This study presents a comprehensive genomic characterization of four Italian sheep breeds using a high-density SNP array. We reported key patterns of genetic diversity, population differentiation, and potential adaptive traits. The results revealed moderate levels of genetic diversity overall, with BAR and NOT exhibiting reduced genetic diversity and elevated inbreeding coefficients, highlighting the urgent need for targeted conservation efforts. The presence, distribution, and characteristics of ROH and HRR reflect the historical breeding and selection processes, which can have both positive and detrimental consequences on genetic diversity and thus the long-term viability of these breeds. Importantly, the identification of ROH and HRR islands harboring genes associated with body weight, milk production, immunity, reproduction, and environmental stress underscores the significant role of selection in shaping genomic architecture of these breeds. Notably, many of the candidate genes highlighted in these regions have been previously reported in other studies as key contributors to important traits. Our VEP analysis corroborated these findings by detecting moderate-impact missense variants within these genes, providing a robust genetic basis for their inclusion in future breed selection and management programs.

A limitation of this study is the relatively small sample size for some of the breeds analyzed, particularly BAR and NOT. This may influence the number of ROH and HRR identified per breed, as breeds with fewer genotyped individuals are likely to yield fewer segments. Therefore, it is difficult to determine whether the observed differences among breeds reflect true genetic characteristics or are partly a consequence of sampling. Future studies with larger sample sizes per breed would help to disentangle these effects and provide more robust estimates of ROH and HRR patterns.

## Supplementary Material

skag014_Supplementary_Data

## Data Availability

The datasets generated and/or analyzed during the current study are not publicly available but are available from the corresponding author upon reasonable request.

## Abbreviations

BAR: Barbaresca
D_HRR_: degree of diversity
DNA: deoxyribonucleic acid
EDTA: ethylenediaminetetraacetic acid
F_IS_: inbreeding coefficient
F_ROH_: inbreeding coefficient calculated on ROH
HD: high-density
H_E_: expected heterozygosity
H_O_: observed heterozygosity
HRR: heterozygosity-rich regions
IBS: identity-by-state
L_HRR_: average length of HRR in Mb per individual
L_ROH_: average length of ROH in Mb per individual
MAF: minor allele frequencies
MDS: multidimensional scaling analysis
N_HRR_: mean number of HRR per individual
NOT: Noticiana
N_ROH_: mean number of ROH per individual
OAR: *Ovis aries* chromosome
ROH: runs of homozygosity
SAR: Sarda
S_HRR_: total length of the genome covered by HRR
SNP: single nucleotide polymorphism
S_ROH_: total length of the genome covered by ROH
VDB: Valle del Belice
VEP: Variant Effect Predictor
