# Supplementary material for: Detection of homozygosity and heterozygosity regions in mediterranean sheep breeds revealed by high-density SNP array
Source: J Anim Sci. 2026 Jan 20;104:skag014. doi: 10.1093/jas/skag014 (PMC12924876; doi:10.1093/jas/skag014)
Supplement: skag014_Supplementary_Data [file skag014_supplementary_data.zip › Table S1.docx]

**Table S1**

Genetic diversity indices calculated for the four breeds. Observed (*H*_O_) and expected (*H*_E_) heterozygosity, inbreeding coefficient (*F*_IS_), and average minor allele frequencies (*MAF*). For all indices, the standard deviation (s.d.) was calculated as well.

| Breed | *H*_O_ | s.d. | *H*_E_ | s.d. | *F*_IS_ | s.d. | *MAF* | s.d. |
| --- | --- | --- | --- | --- | --- | --- | --- | --- |
| BAR | 0.311 | 0.187 | 0.299 | 0.171 | 0.079 | 0.049 | 0.223 | 0.154 |
| NOT | 0.306 | 0.184 | 0.297 | 0.170 | 0.096 | 0.054 | 0.221 | 0.154 |
| SAR | 0.317 | 0.157 | 0.319 | 0.154 | 0.062 | 0.037 | 0.237 | 0.145 |
| VDB | 0.326 | 0.148 | 0.329 | 0.145 | 0.037 | 0.084 | 0.244 | 0.141 |

Abbreviations: BAR = Barbaresca; NOT = Noticiana; SAR = Sarda; VDB = Valle del Belice.
