# Supplementary figures and images for: Detection of homozygosity and heterozygosity regions in mediterranean sheep breeds revealed by high-density SNP array
Source: J Anim Sci. 2026 Jan 20;104:skag014. doi: 10.1093/jas/skag014 (PMC12924876; doi:10.1093/jas/skag014)

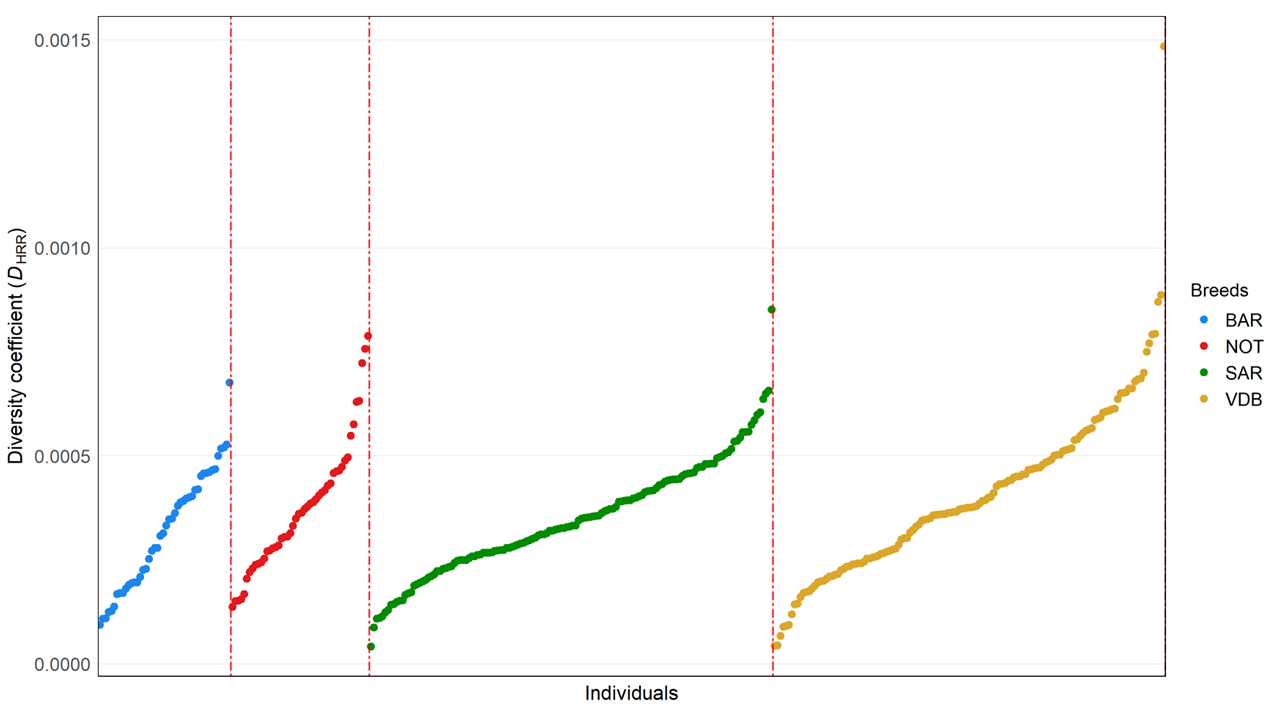

Supplement: skag014_Supplementary_Data [file skag014_supplementary_data.zip › Supplementary Figure S3.tif]

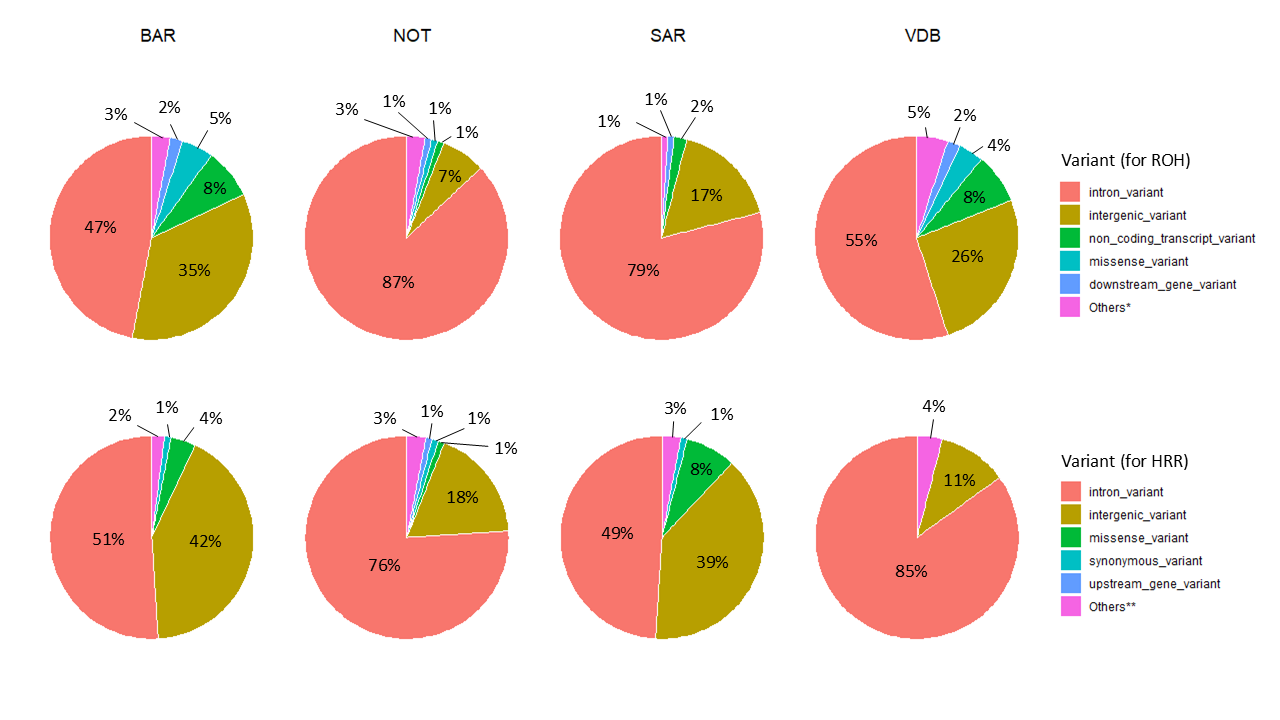

Supplement: skag014_Supplementary_Data [file skag014_supplementary_data.zip › Supplementary Figure S4.tif]

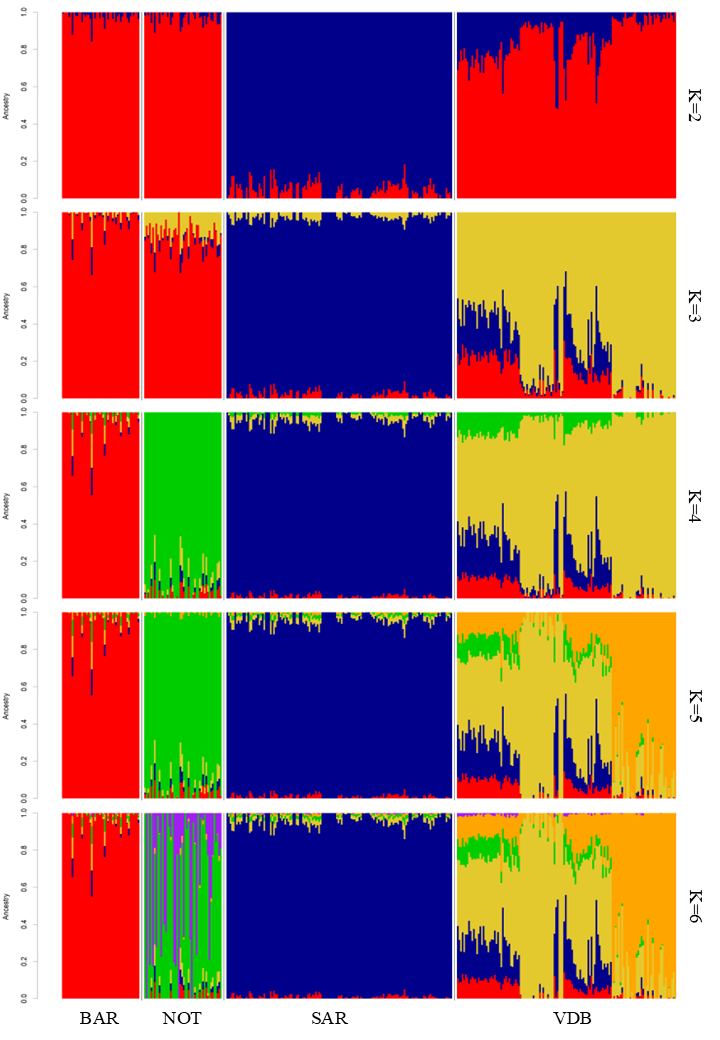

Supplement: skag014_Supplementary_Data [file skag014_supplementary_data.zip › Supplementary Figure S1.tif]

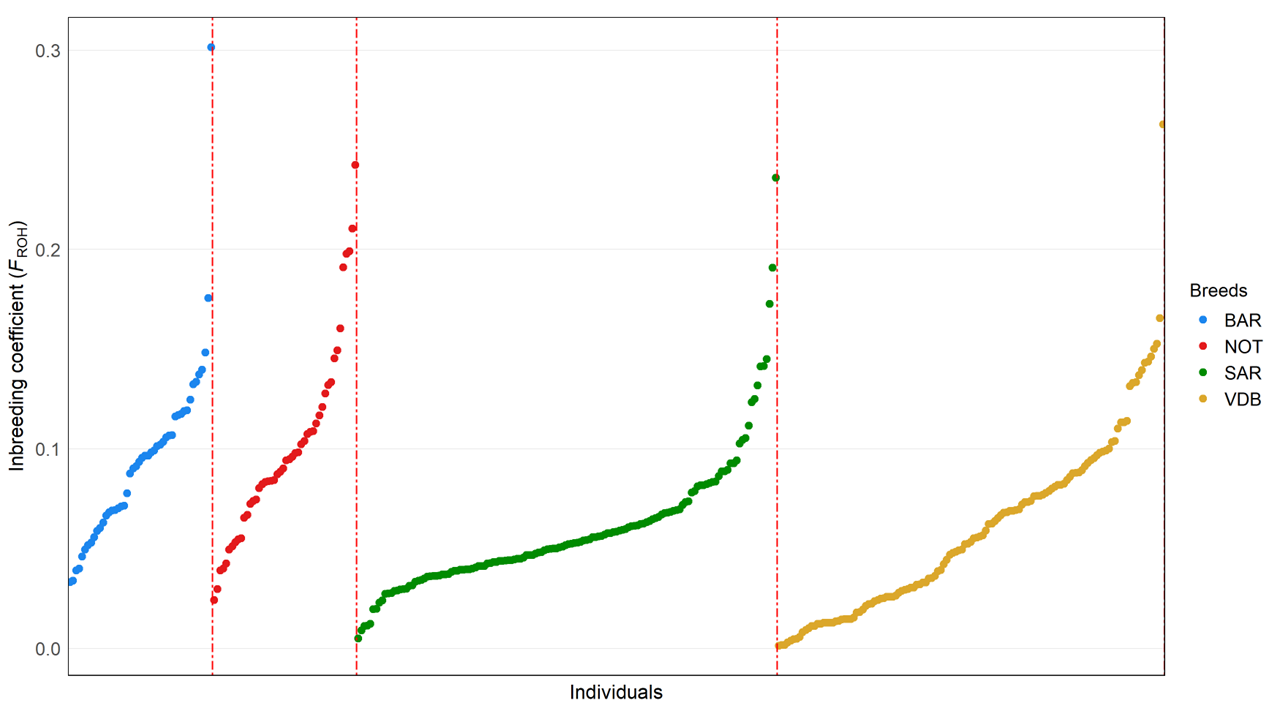

Supplement: skag014_Supplementary_Data [file skag014_supplementary_data.zip › Supplementary Figure S2.tif]
